# Crystal structure of the multiple antibiotic resistance regulator MarR from *Clostridium difficile*

**DOI:** 10.1107/S2053230X1700766X

**Published:** 2017-05-31

**Authors:** J. W. Peng, H. Yuan, X. S. Tan

**Affiliations:** a Department of Chemistry, Fudan University, 220 Handan Road, Shanghai 200433, People’s Republic of China

**Keywords:** *Clostridium difficile*, MarR, crystal structure, transcription factors, DNA binding, antibiotic resistance

## Abstract

Regulators of multiple antibiotic resistance (MarRs) are key players against toxins in prokaryotes. MarR homologues have been identified in many bacterial and archaeal species which pose daunting antibiotic resistance issues that threaten public health. The continuous prevalence of *Clostridium difficile* infection (CDI) throughout the world is associated with the abuse of antibiotics, and antibiotic treatments of CDI have limited effect. In the genome of *C. difficile* strain 630, the *marR* gene (ID 4913953) encodes a MarR protein. Here, MarR from *C. difficile* (MarR*_C.difficile_*) was subcloned and crystallized for the first time. MarR*_C.difficile_* was successfully expressed in *Escherichia coli* in a soluble form and was purified to near-homogeneity (>95%) by a two-step purification protocol. The structure of MarR*_C.difficile_* has been solved at 2.3 Å resolution. The crystal belonged to the monoclinic space group *P*4_3_2_1_2, with unit-cell parameters *a* = *b* = 66.569, *c* = 83.654 Å. The structure reported reveals MarR*_C.difficile_* to be a dimer, with each subunit consisting of six α-helices and three antiparallel β-hairpins. MarR*_C.difficile_* shows high structural similarity to the MarR proteins from *E. coli* and *Staphylococcus aureus*, indicating that MarR*_C.difficile_* might be a DNA-binding protein.

## Abstract 2

The crystal structure of MarR from *C. difficile* is reported.

## 1. Introduction   

*Clostridium difficile* is an anaerobic human pathogen that causes acute healthcare-associated diarrhoea. The morbidity and mortality rates of *C. difficile* infection (CDI) have increased dramatically in Europe and in North America (Heinlen & Ballard, 2010 ▸); the emergence of *C. difficile* strains that are resistant to multiple antibiotic agents can complicate prevention programs and potential treatments (Hunt & Ballard, 2013 ▸). Many studies have demonstrated that various bacterial species employ MarR homologues to sense and exert resistance against many cellular toxins from the environment or host immune system, including multiple antibiotics, oxidative reagents and disinfectants (Cohen *et al.*, 1993 ▸; Alekshun & Levy, 1999 ▸). The regulator of multiple antibiotic resistance (MarR) in *Escherichia coli*, a member of the MarR family of regulator proteins, modulates bacterial detoxification in response to diverse antibiotics (Hao *et al.*, 2014 ▸). The transcription factors of the MarR family regulate diverse genes involved in multiple antibiotic resistance, the synthesis of virulence determinants and many other important biological processes (Martin *et al.*, 1995 ▸; Alekshun & Levy, 1997 ▸; Perera & Grove, 2010 ▸). The MarR protein, as a member of the MarR family of multiple antibiotic resistance proteins, is a key global regulator in *C. difficile*. A link between MarR family proteins and antibiotic resistance has been suggested in previous studies (George & Levy, 1983 ▸). However, the function of MarR in *C. difficile* is still unknown. Here, we aim to study the biological function of MarR from the perspective of its crystal structure. Thus, the major work in this article is to report the crystal structure of MarR from *C. difficile* (MarR*_C.difficile_*).

In this study, we solved the crystal structure of MarR*_C.difficile_* by molecular replacement. Diffraction data were collected to 2.3 Å resolution. The overall structure indicated that MarR*_C.difficile_* is a homodimer, with each subunit consisting of six helical regions and three β-strands. Like other MarR proteins, the helical regions in each subunit contribute to the protein–protein interface in the dimer. An analysis of electrostatic surface potential shows a putative DNA-binding site, as observed in other MarR family proteins. This is the first reported crystal structure of this protein from *C. difficile*.

## 2. Materials and methods   

### 2.1. Protein preparation   

**Figure 1:**
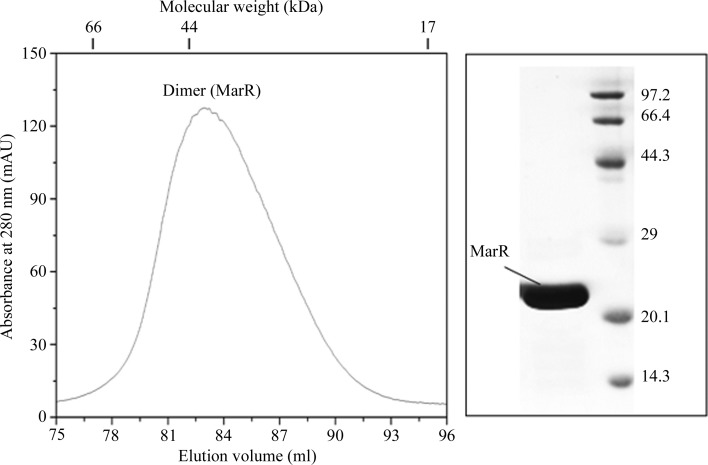
Elution profile of MarR*_C.difficile_* from a Superdex 200 HiLoad 16/60 gel-filtration column on an ÄKTAexplorer FPLC system. The peak at 82 ml (the flow rate was 1 ml min^−1^) represents the MarR*_C.difficile_* dimer. The apparent molecular weight of the eluting species was calculated using standard protein markers (Gel Filtration LMW Calibration Kit, GE Healthcare). The right panel shows SDS–PAGE analysis of the collected fraction.

The gene encoding MarR (annotated in GenBank as CAJ67669.1) was PCR-amplified using *C. difficile* 630 genomic DNA as template, into which NdeI and EcoRI restriction sites were introduced. The purified PCR product was digested with the corresponding restriction enzymes and ligated with T4 DNA ligase into the pET-28a(+) vector (Novagen). The resulting construct contained a hexahistidine tag at the N-terminus of MarR and a thrombin cleavage site. The constructed plasmid was transformed into *E. coli* BL21 cells for expression. The *E. coli* cells were grown in LB medium containing 100 µg ml^−1^ kanamycin at 37°C to an OD_600_ of 0.6 before IPTG was added to a final concentration of 0.4 m*M*. Protein expression was induced at 20°C for 12 h before harvesting. The cell pellets were collected and resuspended in NTA buffer (20 m*M* Tris–HCl pH 7.6, 200 m*M* NaCl, 10% glycerol) containing 1 m*M* phenylmethanesulfonyl fluoride (PMSF). After sonication on ice for 30 min, the cell lysate was spun at 11 000 rev min^−1^ for 30 min. The clear lysate was filtrated and loaded onto a HisTrap column (5 ml column, GE Healthcare), which had been pre-equilibrated with 50 ml NTA buffer (Hao *et al.*, 2014 ▸), for nickel-affinity chromatography. The MarR*_C.difficile_* protein was then eluted with a linear imidazole gradient followed by a further purification step using a HiLoad 16/60 Superdex 200 column (GE Healthcare) equilibrated with buffer *A* (20 m*M* Tris–HCl pH 7.6, 200 m*M* NaCl). The eluted protein was purified to >95% homogeneity as determined by 16% SDS–PAGE analysis (Fig. 1 ▸). The protein was collected and concentrated for crystallization screening.

Information relating to the production of recombinant MarR is summarized in Table 1 ▸.

### 2.2. Crystallization   

Crystals of MarR*_C.difficile_* were grown at 16°C by hanging-drop vapour diffusion. 2 µl purified protein (5 mg ml^−1^) in 200 m*M* NaCl, 20 m*M* Tris pH 7.6 was mixed with 2 µl reservoir buffer [10%(*v*/*v*) 2-propanol, 100 m*M* Tris pH 7.6]. The droplets were equilibrated against 400 µl reservoir buffer. Crystals were looped-out and soaked in cryoprotectant [crystallization buffer containing 20%(*v*/*v*) glycerol] before flash-cooling and storage in liquid nitrogen. Crystallization information is summarized in Table 2 ▸.

### 2.3. Data collection and processing   

X-ray diffraction data were collected to 2.3 Å resolution on beamline BL17U at the Shanghai Synchrotron Radiation Facility (SSRF). Crystals were flash-cooled in mother liquor at the beamline before data collection. All data were processed and reduced using *HKL*-2000 (Otwinowski & Minor, 1997 ▸). The space group of the MarR crystals was determined to be *P*4_3_2_1_2, with one molecule in the asymmetric unit and with unit-cell parameters *a* = *b* = 66.57, *c* = 83.65 Å, α = β = γ = 90° for the native protein. Data-collection and processing statistics are summarized in Table 3 ▸.

### 2.4. Structure solution and refinement   

The structure was solved by molecular replacement using SlyA from *Listeria monocytogenes* (PDB entry 4mnu; Midwest Center for Structural Genomics, unpublished work) as the starting model. The structure was refined using *REFMAC* 5.7.0032 (Winn *et al.*, 2011 ▸; Potterton *et al.*, 2003 ▸; Murshudov *et al.*, 2011 ▸). Finally, the structure was deposited in the Protein Data Bank as PDB entry 5eri. The structure-solution and refinement statistics are summarized in Table 4 ▸ .

## 3. Results and discussion   

### 3.1. Overall structure of MarR*_C.difficile_*   

**Figure 2:**
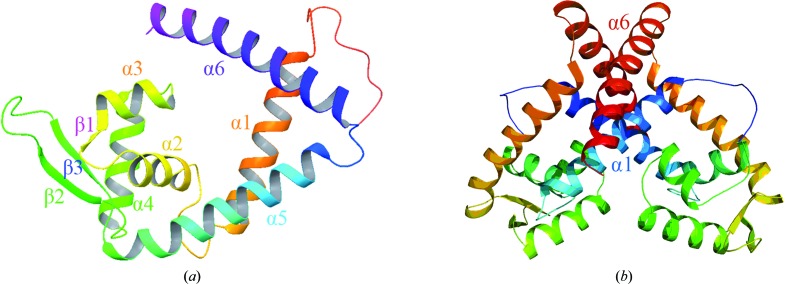
The structure of MarR*_C.difficile_*. (*a*) One MarR*_C.difficile_* subunit with labelled secondary structure. (*b*) Ribbon representation of the crystal structure of the MarR*_C.difficile_* dimer viewed with the subunit twofold axis close to vertical.

The crystal structure of MarR*_C.difficile_* was determined by molecular replacement using SlyA (PDB entry 4mnu) as a search model. Like other MarR proteins, the MarR*_C.difficile_* protein is composed of six α-helices and a three-stranded antiparallel β-hairpin (Fig. 2 ▸ *a*). The α2, α3 and α4 helices and two antiparallel β-strands, β2 and β3, are probably responsible for DNA binding, as indicated by the electrostatic surface potential (Figs. 2 ▸ and 3 ▸). The two putative DNA-binding domains in each subunit result in the formation of a channel through the centre of the dimer (Figs. 2 ▸ and 3 ▸). Consistent with previous studies, the putative DNA-binding regions of the MarR*_C.difficile_* protein are strongly electropositive, as are other winged-helix DNA-binding proteins (Gajiwala & Burley, 2000 ▸).

The structure of MarR from *E. coli* (MarR*_E.coli_*) is a homodimer (Alekshun *et al.*, 2001 ▸), and it has been verified that MarR*_E.coli_* binds the *marRAB* promoter as a dimer (Martin *et al.*, 1996 ▸). A recent study showed that disulfide bonds could be formed between MarR*_E.coli_* dimers, resulting in the dissociation of MarR*_E.coli_* from its cognate DNA and enhanced bacterial resistance (Zhu *et al.*, 2017 ▸). The MarR family member MprA also functions as a dimer (Brooun *et al.*, 1999 ▸). *PISA* analysis suggested that MarR*_C.difficile_* is a homodimer. In the crystal structure of MarR*_C.difficile_* there is one monomer in the asymmetric unit, with the dimer being composed of two subunits related by a crystallographic twofold rotation. The crystal structure also indicates that α-helices in the N- and C-terminal regions of each subunit interdigitate with those of the other subunit to form a hydrophobic core burying a surface area of 3100 Å^2^. Two helical regions, α1 and α6 (residues 9–27 in the N-terminus and residues 123–147 in the C-terminus, respectively), are closely juxtaposed and intertwine with the equivalent regions of the second subunit to form a dimer. The dimer is stabilized by several salt bridges, notably that between Arg16 and Glu74′ and that between Lys155 and Glu145′. In addition, a hydrogen bond between the side-chain carbonyl O atom of Glu66 and the guanidinium NH groups of Arg31′ enhances the stability of the dimeric structure.

### 3.2. Comparison of MarR*_C.difficile_* with MarR*_E.coli_* and MgrA*_S.aureus_*   

MgrA from *Staphylococcus aureus* (MgrA*_S.aureus_*) is a regulator of antibiotic resistance and is also an important virulence determinant during infection (Ingavale *et al.*, 2005 ▸). A previous study indicated that the cysteine residue (Cys12) of this protein could be oxidized by various reactive oxygen species. Cysteine oxidation leads to the dissociation of MgrA from DNA, resulting in the initiation of signalling pathways and further enhancing antibiotic resistance in *S. aureus* (Chen *et al.*, 2006 ▸). The oxidation-sensing mechanism is widely used by bacteria to counter challenges of environmental pressure (Lee & Helmann, 2006 ▸). In conclusion, MgrA from *S. aureus* is an oxidation sensor.

Previous studies reported that the MarR family of proteins are typically conserved transcription factors that modulate bacterial resistance to multiple antibiotics, oxidative reagents and detergents (Martin & Rosner, 1995 ▸). Various bacterial species such as *E. coli* can respond to environmental stresses such as toxic chemicals and disinfectants by triggering the dissociation of MarR from the cognate DNA in a copper-dependent manner. The detailed mechanism is that copper(II) oxidizes a unique cysteine residue (Cys80) that resides in the DNA-binding domain of MarR*_E.coli_* to generate inter-dimer disulfide bonds, thereby inducing tetramer formation and the dissociation of MarR from the *marRAB* promoter (Hao *et al.*, 2014 ▸). Therefore, MarR from *E. coli* is a copper signal oxidation sensor.

**Figure 4:**
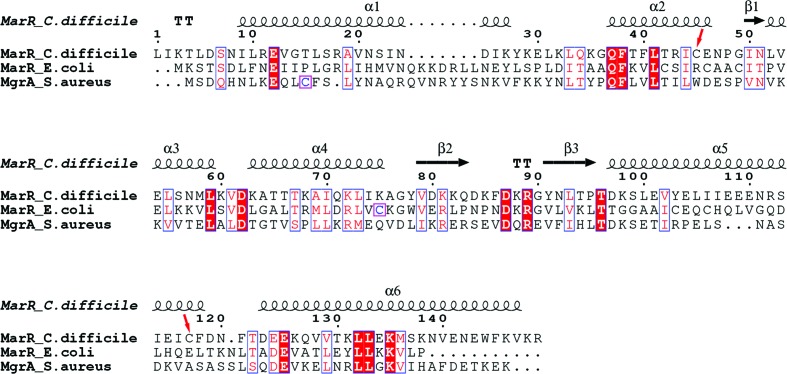
Primary-sequence alignment of MarR*_C.difficile_* with representative members of the MarR family (MarR*_E.coli_* and MgrA*_S.aureus_*). The secondary-structural elements of MarR*_C.difficile_* are indicated above the sequence alignment: α-helices (α) are illustrated as curly lines and arrows represent β-sheets (β). Numbering is according to the MarR*_C.difficile_* primary sequence. Residues that are identical in all homologues are highlighted in red and highly conserved amino acids are shown in blue boxes. Red arrows indicate the cysteine residues of MarR*_C.difficile_*; the key cysteine residues of MarR*_E.coli_* and MgrA_*S.aureus*_ are shown in purple boxes.

**Figure 5:**
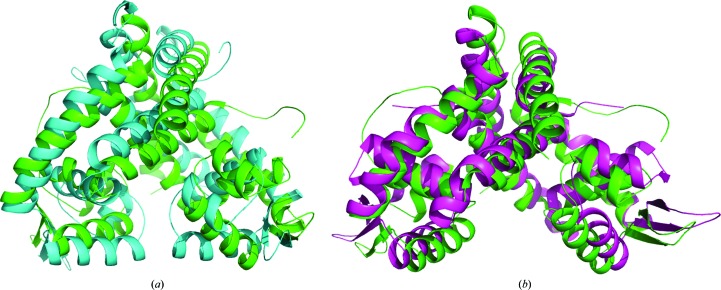
Superimposition of MarR*_C.difficile_* with MarR*_E.coli_* and MgrA*_S.aureus_*. (*a*) MarR*_C.difficile_* aligned with MarR*_E.coli_* (MarR*_C.difficile_*, green; MarR*_E.coli_*, cyan). (*b*) MarR*_C.difficile_* aligned with MgrA*_S.aureus_* (MarR*_C.difficile_*, green; MgrA*_S.aureus_*, purple).

 Sequence alignment of MarR*_C.difficile_* with MarR*_E.coli_* and MgrA*_S.aureus_* using *MUSCLE* shows 26 and 19% sequence identity; these proteins share low sequence similarity (Fig. 4 ▸). However, superposition of MarR*_C.difficile_* with MgrA*_S.aureus_* (PDB entry 2bv6; Chen *et al.*, 2006 ▸) and MarR*_E.coli_* (PDB entry 1jgs; Alekshun *et al.*, 2001 ▸) shows structural similarity (Fig. 5 ▸). The MgrA*_S.aureus_* dimer is triangular in shape, with two winged-helix DNA-binding domains; the DNA-binding domain includes two α-helices, two β-sheets and a wing region (Chen *et al.*, 2006 ▸). The structure of MarR*_E.coli_* is a crystallographic dimer, with each subunit containing a winged-helix DNA-binding motif, and this DNA-binding motif contains three α-helices and two β-strands (Alekshun *et al.*, 2001 ▸). We found that the crystal structure reveals MarR*_C.difficile_* to be a dimer, with each monomer consisting of six α-helices and a three-stranded antiparallel β-hairpin. The putative DNA-binding domain of each subunit includes three α-helices and two antiparallel β-strands. Correspondingly, MarR*_E.coli_* and MgrA*_S.aureus_* share a similar oxidation-sensing mechanism in which cysteine oxidation leads to the dissociation of MarR*_E.coli_* and MgrA*_S.aureus_* from DNA. As a result, they exhibit a similar function. However, the function of MarR in *C. difficile* remains unknown. Structural analysis of MarR*_C.difficile_* indicated that two cysteine residues (Cys45 and Cys117) are located in the hydrophobic core; this may suggest that MarR in *C. difficile* is probably not an oxidation sensor. Although they share structural similarity, these proteins might have diverse molecular mechanisms.

## 4. Conclusion   

Although the MarR protein has been well studied in many species, the MarR protein from *C. difficile* remains unknown. The relationship between MarR and antibiotic resistance in *C. difficile* needs to be investigated. The crystal structure reported in this paper reveals MarR*_C.difficile_* to be a crystallo­graphic dimer. It shows structural similarity to other MarR family proteins. Furthermore, the structure of MarR in *C. difficile* suggests a putative DNA-binding site, revealing that the MarR protein in *C. difficile* might be also a transcription factor that can bind DNA. Based on the structural analysis of MarR*_C.difficile_*, we found that the two cysteine residues could not be oxidized easily as they are located in the hydrophobic core. Therefore, MarR from *C. difficile* may not be an oxidation sensor. The solved crystal structure of MarR*_C.difficile_* will be the first step in further functional studies.

## Supplementary Material

PDB reference: MarR, 5eri

## Figures and Tables

**Figure 3:**
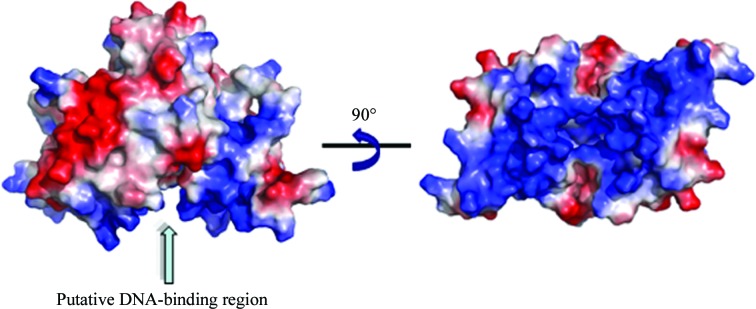
Electrostatic surface representation of the MarR*_C.difficile_* dimer. The putative DNA-binding sites are indicated by an arrow.

**Table 1:** Information relating to the production of recombinant MarR

Source organism	*C. difficile*
DNA source	*C. difficile* strain 630 genomic DNA
Forward primer†	5′-GCGCGCGGGAATTCCATATGTTGATTAAGACTTTAGATAGTAATATATTAAGAG-3′
Reverse primer‡	5′-GCCCGGAATTCCTATCTCTTTACTTTAAACCATTCATTTTCTACG-3′
Cloning vector	pET-28a(+) (Novagen)
Expression vector	pET-28a(+) (Novagen)
Expression host	*E. coli* BL21
Complete amino-acid sequence of the construct produced§	MGSSHHHHHHSSGLVPRGSHMLIKTLDSNILREVGTLSRAVNSINDIKYKELKLQKGQFTFLTRICENPGINLVELSNMLKVDKATTTKAIQKLIKAGYVDKKQDKFDKRGYNLTPTDKSLEVYELIIEEENRSIEICFDNFTDEEKQVVTKLLEKMSKNVENEWFKVKR

† The NdeI site for cloning is underlined.

‡ The EcoRI site for cloning is underlined.

§ The N-terminal hexahistidine tag, linker and thrombin cleavage site are underlined.

**Table 2:** Crystallization

Method	Vapour diffusion, hanging drop
Plate type	24-well
Temperature (K)	289
Protein concentration (mg ml^−1^)	5
Buffer composition of protein solution	20 m*M* Tris pH 7.6, 200 m*M* NaCl
Composition of reservoir solution	10% 2-propanol, 0.1 *M* Tris–HCl pH 7.6
Volume and ratio of drop	4 µl, 1:1
Volume of reservoir (ml)	0.4

**Table 3:** Data collection and processing

Diffraction source	Beamline BL17U, SSRF
Wavelength (Å)	0.988
Temperature (K)	100
Detector	ADSC 315r
Crystal-to-detector distance (mm)	250
Rotation range per image (°)	1
Total rotation range (°)	180
Exposure time per image (s)	1
Space group	*P*4_3_2_1_2
*a*, *b*, *c* (Å)	66.57, 66.57, 83.65
α, β, γ (°)	90, 90, 90
Mosaicity (°)	0.520
Resolution range (Å)	50–2.297 (2.34–2.30)
Total No. of reflections	207959
No. of unique reflections	8352
Completeness (%)	93.8 (98.8)
Multiplicity	24.9 (28.5)
〈*I*/σ(*I*)〉	77.0 (16.01)
*R*_merge_	0.080 (0.530)
*R*_r.i.m._	0.087 (0.604)
*R*_p.i.m._	0.019 (0.110)
Overall *B* factor from Wilson plot (Å^2^)	48.6

**Table 4:** Structure solution and refinement

Resolution range (Å)	25.73–2.30 (2.357–2.297)
Completeness (%)	93.8 (98.8)
σ Cutoff	*I* > 3σ(*I*)
No. of reflections, working set	7915 (586)
No. of reflections, test set	394 (26)
Final *R*_cryst_	0.212 (0.280)
Final *R*_free_	0.250 (0.318)
Cruickshank DPI	0.337
No. of non-H atoms	
Protein	1246
Ligand	0
Water	33
Total	1279
R.m.s. deviations	
Bonds (Å)	0.019
Angles (°)	1.789
Average *B* factors (Å^2^)	
Protein	59.9
Water	56.2
Ramachandran plot	
Most favoured (%)	99
Allowed (%)	1
